# Required warfarin dose and time in therapeutic range in patients with diagnosed Nonalcoholic Fatty Liver Disease (NAFLD) or Nonalcoholic Steatohepatitis (NASH)

**DOI:** 10.1371/journal.pone.0251665

**Published:** 2021-09-15

**Authors:** Xuerong Wen, Shuang Wang, Tracey H. Taveira, Fatemeh Akhlaghi

**Affiliations:** 1 Health Outcomes, Department of Pharmacy Practice, College of Pharmacy, University of Rhode Island, Kingston, RI, United States of America; 2 Cardiovascular Department, Providence Veterans Affairs Medical Center, Providence, RI, United States of America; 3 Warren Alpert School of Medicine, Brown University, Providence, RI, United States of America; 4 Clinical Pharmacokinetics Research Laboratory, Department of Biomedical and Pharmaceutical Science, College of Pharmacy, University of Rhode Island, Kingston, RI, United States of America; Ohio State University, UNITED STATES

## Abstract

Warfarin has been widely used to treat thromboembolism. The effect of nonalcoholic fatty liver disease (NAFLD) or nonalcoholic steatohepatitis (NASH), on warfarin dosing remains unknown. This study aims to examine the effects of NAFLD/NASH on the average daily dose (ADD) of warfarin and the time in therapeutic range (TTR). This is a retrospective study utilizing an administrative data. We included patients with at least 2 months of warfarin dispensing and two subsequent consecutive INR measures. The ADD of warfarin to achieve therapeutic range INR levels, and TTR were compared between patients with and without NAFLD/NASH in four subgroups of patients accounting for the presence of obesity and diabetes. Generalized linear models (GLM) with Propensity score (PS) fine stratification were applied to evaluate the relative differences (RD) of warfarin ADD and TTR (>60%) in four subgroups. A total of 430 NAFLD/NASH patients and 38,887 patients without NAFLD/NASH were included. The ADD and TTR, were not significant in the overall cohort between those with and without NAFLD/NASH. However, GLM results in patients without diabetes or obesity (N = 26,685) showed a significantly lower warfarin ADD (RD: -0.38; 95%CI: -0.74–-0.02) and shorter TTR (OR: 0.71; 95%CI: 0.52–0.97) in patients diagnosed with NAFLD/NASH. The effects of NAFLD/NASH on warfarin dose or TTR were observed in patients without obesity and diabetes. Obesity and diabetes appear to be significant modifiers for the effects of NAFLD/NASH on warfarin dose and TTR.

## Introduction

Warfarin is the most commonly used oral anticoagulant in the United States (US) [1]. It is used to prevent and treat thromboembolism in patients with chronic atrial fibrillation, prosthetic heart valves, and venous thromboembolism [2]. Guidelines recommend long-term warfarin use with a dose adjustment to a target international normalized ratio (INR) range of 2.0 to 3.0 for most indications [3,4]. Subtherapeutic INR may increase the risk of ischemic stroke, while supratherapeutic INR may lead to major bleeding, including intracranial hemorrhage [5,6].

Although estimation of the warfarin dose with clinical and pharmacogenetic data has been well developed [7–10], INR control and warfarin dose prediction are still challenging in clinical practice due to a narrow therapeutic window, variable pharmacokinetic profiles, and multiple drug-drug, drug-disease, and drug-food interactions [11–14]. To improve the quality of warfarin therapy, clinical guidelines have been provided pertaining to patient selection, warfarin initiation, dose optimization, drug interactions, switching among anticoagulants, and etc. [15]. Some health providers in the USA have offered specialized anticoagulation clinics exclusively for the management of warfarin dosing [16]. It has been noted that obese and morbidly obese patients required a higher average daily dose (ADD) compared to patients with non-obese [17]. Although liver disease is a significant risk factor for hemorrhage in warfarin users [18,19], the impact of nonalcoholic fatty liver disease (NAFLD) or nonalcoholic steatohepatitis (NASH) on warfarin dosing and time in therapeutic range (TTR) remains unclear.

NAFLD/NASH has become the most common chronic liver disease that affects approximately 30 million people in the US [20,21]. As reviewed in Cobbina et al., NAFLD/NASH may decrease the expression and activity of several cytochrome P450 (CYP) enzymes responsible for the metabolism of warfarin [22]. Moreover, procoagulant imbalance may occur in patients with NAFLD/NASH [23]. Previous studies have demonstrated that NAFLD/NASH is independently associated with an increased risk of atrial fibrillation (AF) in adults and elderlies, especially those with type 2 diabetes mellitus (T2DM) [24,25]. Evidence also suggest a higher rate of unprovoked venous thromboembolism (VTE) in patients with NAFLD/NASH [26–28]. Given this strong relationship between NAFLD/NASH and AF and VTE, more patients with NAFLD/NASH will be affected by cardiovascular diseases and treated with anticoagulants.

Direct oral anticoagulants (DOACs) have become the first-line anticoagulant treatment for patients with non-valvular AF or VTE due to their improved efficacy/safety profiles and fewer follow-up monitoring requirements compared to warfarin [29,30]. The use of DOACs is exceeding that of warfarin even in liver disease patients since real-world data studies suggest that DOACs provide non-inferior efficacy and improved safety in liver disease patients as compared to those without liver disease [31–33] However, historical cohorts of patients with liver diseases, including NAFLD/NASH, are still anticoagulated with warfarin.

Therefore, we are motivated to conduct a population-based study to investigate the impact of NAFLD/NASH on therapeutic warfarin dose and the associated anticoagulation control among stabilized warfarin users. Our primary aim was to evaluate the effects of diagnosed NAFLD/NASH on warfarin dosing and time in therapeutic range.

## Materials and methods

### Data sources

Data were obtained from Optum from January 1, 2010 to September 30, 2015. The Optum’s deidentified Clinformatics® Data Mart Database is a large Commercial and Medicare Advantage claims database from a large national insurer that includes longitudinal health data of 35 million individuals enrolled in private and Medicare health plans from 2010-2015 [34]. This study was approved as an exempted study by the Institutional Review Board of University of Rhode Island (IRB#: 1423847-1).

### Study cohort

**Fig 1:**
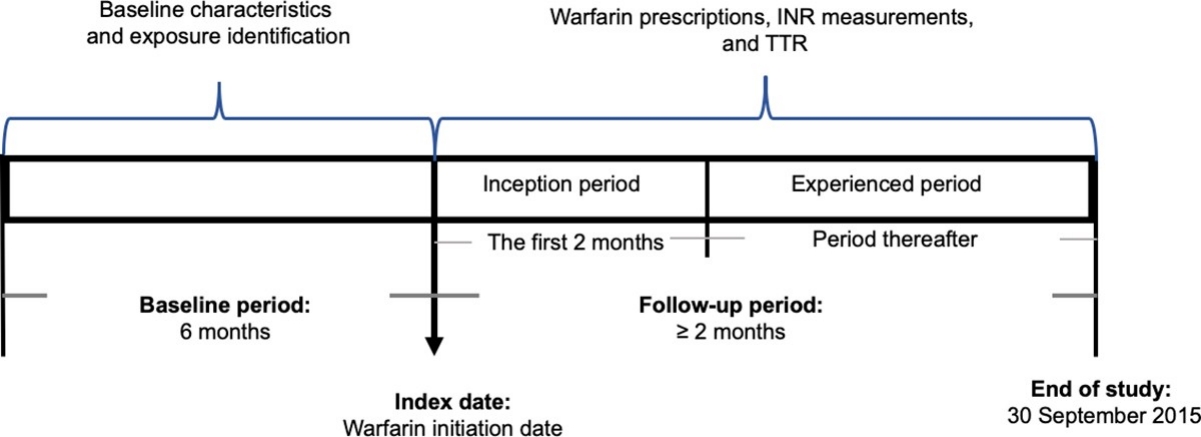
Schematic diagram for the study design. Note: INR: International Normalized Ratio, TTR: Time to Therapeutic Range.

This is a retrospective cohort study based on the administrative claims data. Fig 1 presents the schematic plot of the study design. The study cohort consisted of adults (aged 18 years or older as of their warfarin initiation date) with at least 2 months of continuous warfarin dispensing and 2 consecutive INR measurements during the warfarin therapy starting from July 1, 2010. The first warfarin prescription dispensing date was defined as the index date. The baseline period was defined as 6 months prior to the index date. Patients were followed from the index date until the end of available warfarin pharmacy claims or INR records or September 30, 2015, whichever occurred first (Fig 1).

Participants were required to be continuously enrolled in a health plan during the baseline and follow-up periods. Patients with one or more diagnoses indicating hepatitis B virus infection (HBV), hepatitis C virus infection (HCV), alcoholic liver disease or alcohol use disorder during the baseline period were excluded. INR measurements were excluded if more than two INR measurements were recorded on the same day for one patient. Based on the clinical practice of warfarin prescribing, we excluded warfarin prescriptions if the computed daily dose exceeded 50 mg/d.

### Definition of exposure

Participants who had at least two outpatient visits or one admission claim with an ICD-9-CM code for NAFLD/NASH (571.8 for other chronic non- alcoholic liver disease, 571.9 for unspecified chronic liver disease without mention of alcohol, or 571.5 for cirrhosis of the liver without mention of alcohol) during the baseline period were identified as the exposed group [35]. Due to the tendency of underestimating NAFLD/NASH in a claims database, we included cirrhosis of liver without mention of alcohol after excluding alcohol related liver disease and use disorder. Participants who had no diagnosis of NAFLD/NASH during the baseline period were considered the reference group.

### Covariates

Covariates were assessed in the 6 months preceding the index date. Baseline demographics included age (as of warfarin initiation date), gender and insurance type. A medical history of diabetes mellitus, obesity, hypertension, chronic kidney disease, dementia, hyperlipidemia, heart failure, atrial fibrillation, valvular heart disease, and coronary heart disease were also included as potential confounding variables [36,37]. Data involving psychiatric diagnoses such as depression, anxiety or post-traumatic stress disorder (PTSD), and psychosis were also abstracted. Patients co-medication use was controlled and defined as prescriptions filled during the 3 months before the index date and included antibiotics, nonsteroidal anti-inflammatory drugs (NSAIDs), selective serotonin reuptake inhibitors (SSRIs), antithrombotic agents, antiulcer agents and acid suppressants, antihyperglycemics, and antihypertensives [37–41].

### Outcome assessments

The primary outcome was defined as an ADD of warfarin. Warfarin use was determined by the presence of ≥ 1 warfarin prescription claim. Detailed information, such as fill date, strength, days of supply and quantity were collected in Optum database. The ADD of warfarin was calculated using the following formula: (strength*quantity)/days of supply. If a patient had multiple prescriptions on the same day, the formula of daily dose = sum(strength*quantity) / max(days of supply) was used. The ADD that exceeded 50 mg was eliminated as it was likely to be a data entry error and may become an influential outlier that biased the results.

The secondary outcome was defined as TTR>60%, which indicates a high-quality anticoagulation control as per previous studies [42,43]. TTR was calculated according to the Rosendaal method, in which linear interpolation was used to assign an INR value to each day between two consecutively measured INR values [44]. The time spans for INRs with ≥56 days between 2 successive measurements were excluded from the TTR calculation. The TTR was calculated as the days with INR values between 2 and 3 divided by overall days between all successive INR measures that were included. We defined the INR stabilization as the first 3 consecutive INR values within 2.0 and 3.0 after warfarin initiation [45].

### Statistical analysis

Continuous variables are presented as mean (SD), and compared using the independent t test between patients with and without NAFLD/NASH. Categorical variables are reported as frequency (percentage) and compared using chi-square test between patients with and without NAFLD/NASH. Propensity score fine stratification is applied to adjust for confounding and to achieve comparability among the exposed and reference groups [46]. More specifically, we adopted the PS fine stratification exposure approach in which matching mechanism is based on the exposed group instead of the entire cohort, to address the infrequent prevalence of NAFLD/NASH (~1%) in our study cohort [29]. We selected either 50 or 100 strata depending the subgroup sample size. We calculated standardized difference to assess balance between NAFLD/NASH cohort and the non-NAFLD/NASH cohort.

For outcome models, we applied generalized linear model (GLM) with PS fine stratification to evaluate the effect of NAFLD/NASH on warfarin dose and TTR in patients with a therapeutic INR. In addition, we included interaction terms between NAFLD/NASH, obesity and diabetes mellitus in the analysis to examine the modified association. In the secondary analysis, we conducted subgroup analysis among a subset of patients stratified by their obesity and diabetes status using GLM with PS fine stratification. All analyses were conducted using SAS 9.4 (SAS Inc., Cory, NC). A statistically significant level was set up at p<0.05.

## Results

**Fig 2:**
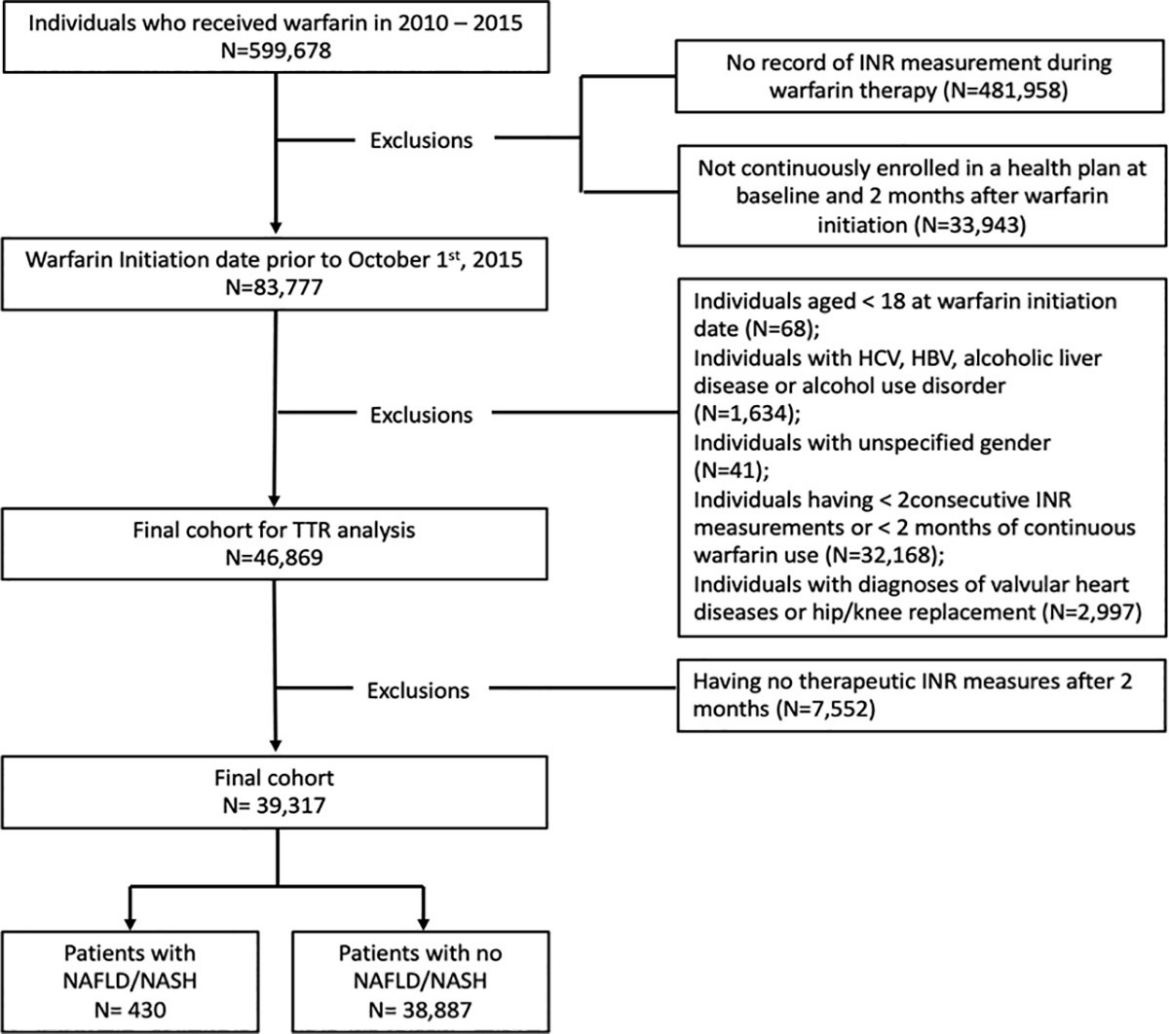
Flow chart of study cohort selection. Note: INR: International Normalized Ratio, TTR: Time to Therapeutic Range; NAFLD: Nonalcoholic Fatty Liver Disease; NASH: Nonalcoholic Steatohepatitis; HBV: Hepatitis B Virus Infection, HCV: Hepatitis C Virus Infection.

Fig 2 shows the flow chart of study inclusion/exclusion, and assembling of the final study cohort. After excluding patients who did not have enough baseline period, records of INR measurements, or continuous warfarin dispensing for 2 months, there were 83,777 patients who received warfarin prior to October 1, 2015. We excluded 1,634 participants with 1 or more diagnoses of HBV, HCV, alcoholic liver disease or alcohol use disorder, 2,997 with diagnoses of valvular heart diseases or hip/knee replacement, 68 non-adults, and 32,209 having < 2 months of continuous warfarin use or <2 consecutive INR measurements. A total 39,317 patients were included in the final cohort for warfarin dose analysis, including 430 patients diagnosed with NAFLD/NASH and 38,887 patients without NAFLD/NASH.

In this study, the mean warfarin ADD in patients diagnosed with NAFLD/NASH was 5.853 mg, and 5.848 mg in patients without NAFLD/NASH. We identified 36.72% (n = 384) of NAFLD/NASH patients with TTR>60%, and 45.25% (n = 35,381) of patients without NAFLD/NASH have TTR>60%.

Table 1 presents demographic and clinical characteristics of the study cohort before propensity score fine stratification. The age distribution reflects the fact that the indication for warfarin is the most common in the elderly. Patients diagnosed with NAFLD/NASH had a considerably higher risk of comorbidities. Of 430 NAFLD/NASH patients, 39.1% had diabetes and 26.7% had obesity, which are considerably higher than patients without NAFLD/NASH. Other comorbidities that are greater in NAFLD/NASH patients included myocardial infarction, non-valvular atrial fibrillation, chronic renal disease, hypertension, hyperlipidemia, heart failure, valvular heart disease, coronary heart disease, depression, or psychosis. Furthermore, patients diagnosed with NAFLD/NASH more frequently received co-medications, including antibiotics, NSAIDs, antithrombotic agents, SSRIs, antiulcer agents and acid suppressants, and antidiabetic agents.

**Table 1:** Demographic and clinical characteristics of study population selected from health plan data between 2010 – 2015.

Characteristics	Patients with NAFLD/NASH diagnosis N = 430	Patients without NAFLD/NASH diagnosis N = 38887	Standardized difference,%	P-value
**Age category, n (%)**				
< 65	194 (45.1)	8890 (22.9)	48.3	<.0001
65-75	139 (32.3)	10314 (26.5)	12.8	<.0001
75-85	88 (20.5)	15442 (39.7)	-42.9	<.0001
>85	9 (2.1)	4241 (10.9)	-36.3	<.0001
**Health plan**				
HMO	140 (32.6)	19977 (51.4)	-38.8	<.0001
PPO	45 (10.5)	2576 (6.6)	13.8	<.0001
Other	245 (57)	16334 (42)	30.3	<.0001
**Gender, Male**	230 (53.5)	20323 (52.3)	2.5	0.6125
**Indication, n (%)**				
Myocardial infarction	123 (28.6)	7732 (19.9)	20.5	<.0001
non-valvular atrial fibrillation	265 (61.6)	20976 (53.9)	15.6	<.0001
Others	42 (9.8)	10179 (26.2)	-43.7	<.0001
**Co-medication, n (%)**				
Antibiotics	167 (38.8)	10406 (26.8)	25.9	<.0001
NSAIDs	45 (10.5)	2585 (6.6)	13.7	0.0016
Antithrombotic agents	128 (29.8)	5564 (14.3)	38	<.0001
SSRI	68 (15.8)	4350 (11.2)	13.6	0.0025
Antiulcer/Acid Suppressants	153 (35.6)	8266 (21.3)	32.2	<.0001
Antidiabetics	135 (31.4)	8010 (20.6)	24.8	<.0001
Antihypertensive	321 (74.7)	30512 (78.5)	-9	0.056
**Chronic medical condition, n (%)**				
Diabetes	168 (39.1)	8059 (20.7)	40.9	<.0001
Obesity	115 (26.7)	3040 (7.8)	51.7	<.0001
Chronic renal disease	122 (28.4)	5782 (14.9)	33.3	<.0001
Hypertension	335 (77.9)	22947 (59)	41.5	<.0001
Hyperlipidemia	274 (63.7)	18064 (46.5)	35.3	<.0001
Heart Failure	134 (31.2)	8114 (20.9)	23.6	<.0001
Atrial fibrillation	190 (44.2)	18998 (48.9)	-9.4	0.0541
Valvular heart disease	113 (26.3)	7217 (18.6)	18.6	<.0001
Coronary Heart disease	148 (34.4)	9900 (25.5)	19.7	<.0001
Dementia	15 (3.5)	1151 (3)	3	0.5205
Anxiety/PTSD	58 (13.5)	2214 (5.7)	26.7	<.0001
Depression	77 (17.9)	3110 (8)	29.8	<.0001
Psychosis	35 (8.1)	1475 (3.8)	18.4	<.0001

Note: NAFLD: Nonalcoholic fatty liver disease; NASH: Nonalcoholic Steatohepatitis; HMO: Health maintenance organization; PPO: Preferred provider organization; NSAIDs: Nonsteroidal anti-inflammatory drugs; SSRI: Selective serotonin reuptake inhibitors; PTSD: Post-traumatic stress disorder.

Table 2 shows two groups were well balanced on all covariates after adjusting by propensity score fine stratification. P value are large for each comparison.

**Table 2:** Demographic and clinical characteristics using propensity score fine stratification, USA 2010 – 2015.

Characteristics	Patients with NAFLD/NASH N = 429	Patients without NAFLD/NASH N = 35523	Standardized difference, %†	P-value
**Age category, n (%)**				
< 65	193 (45.1)	15943 (44.9)	0.4	0.8646
65-75	139 (32.5)	11985 (33.7)	-2.7	0.8646
75-85	88 (20.6)	7014 (19.7)	2	0.8646
>85	8 (1.9)	579 (1.6)	1.8	0.8646
**Health plan**				
HMO	138 (32.2)	11360 (32)	0.6	0.9458
PPO	45 (10.5)	3752 (10.6)	-0.2	0.9458
Other	245 (57.2)	20410 (57.5)	-0.4	0.9458
**Gender, Male**	230 (53.7)	19100 (53.8)	-0.1	0.9641
**Indication, n (%)**				
Myocardial infarction	121 (28.3)	9954 (28)	0.6	0.858
non-valvular atrial fibrillation	265 (61.9)	22354 (62.9)	-2.1	0.858
Others	42 (9.8)	3213 (9)	2.6	0.858
**Co-medication, n (%)**				
Antibiotics	165 (38.6)	13665 (38.5)	0.2	0.9391
NSAIDs	45 (10.5)	3669 (10.3)	0.6	0.8488
Antithrombotic agents	127 (29.7)	10701 (30.1)	-1	0.9757
SSRI	68 (15.9)	5581 (15.7)	0.5	0.8198
Antiulcer/Acid Suppressants	152 (35.5)	12520 (35.2)	0.6	0.8678
Antidiabetics	135 (31.5)	10967 (30.9)	1.4	0.8005
Antihypertensive	320 (74.8)	26443 (74.4)	0.7	0.8942
**Chronic medical condition, n (%)**				
Diabetes	167 (39)	13819 (38.9)	0.2	0.9297
Obesity	114 (26.6)	9047 (25.5)	2.7	0.6031
Chronic renal disease	121 (28.3)	9986 (28.1)	0.3	0.9827
Hypertension	333 (77.8)	27829 (78.3)	-1.3	0.7895
Hyperlipidemia	273 (63.8)	22751 (64)	-0.5	0.7831
Heart Failure	133 (31.1)	10968 (30.9)	0.4	0.9164
Atrial fibrillation	189 (44.2)	15806 (44.5)	-0.7	0.9495
Valvular heart disease	111 (25.9)	9143 (25.7)	0.4	0.8886
Coronary Heart disease	148 (34.6)	12239 (34.5)	0.3	0.9964
Dementia	15 (3.5)	1273 (3.6)	-0.4	0.9635
Anxiety/PTSD	58 (13.6)	4639 (13.1)	1.4	0.7893
Depression	76 (17.8)	6199 (17.5)	0.8	0.911
Psychosis	34 (7.9)	2887 (8.1)	-0.7	0.8517

Note: NAFLD: Nonalcoholic fatty liver disease; NASH: Nonalcoholic Steatohepatitis; HMO: Health maintenance organization; PPO: Preferred provider organization; NSAIDs: Nonsteroidal anti-inflammatory drugs; SSRI: Selective serotonin reuptake inhibitors; PTSD: Post-traumatic stress disorder; †: The unit of the standardized difference is %. After Propensity Score fine stratification, each standardized difference is less than 3%. P value for each comparison is very large, which means the comparison groups are balanced.

Using GLM with PS fine stratification with main effects only (no interaction terms), no significant effect of NAFLD/NASH was observed on either warfarin dose or TTR>60% (S1 Table). While obesity is associated with an increased warfarin dose, diabetes is related to a decreased warfarin dose (S1 Table). Both obesity and diabetes are related to decreased odds of having TTR>60% (S1 Table).

**Fig 3:**
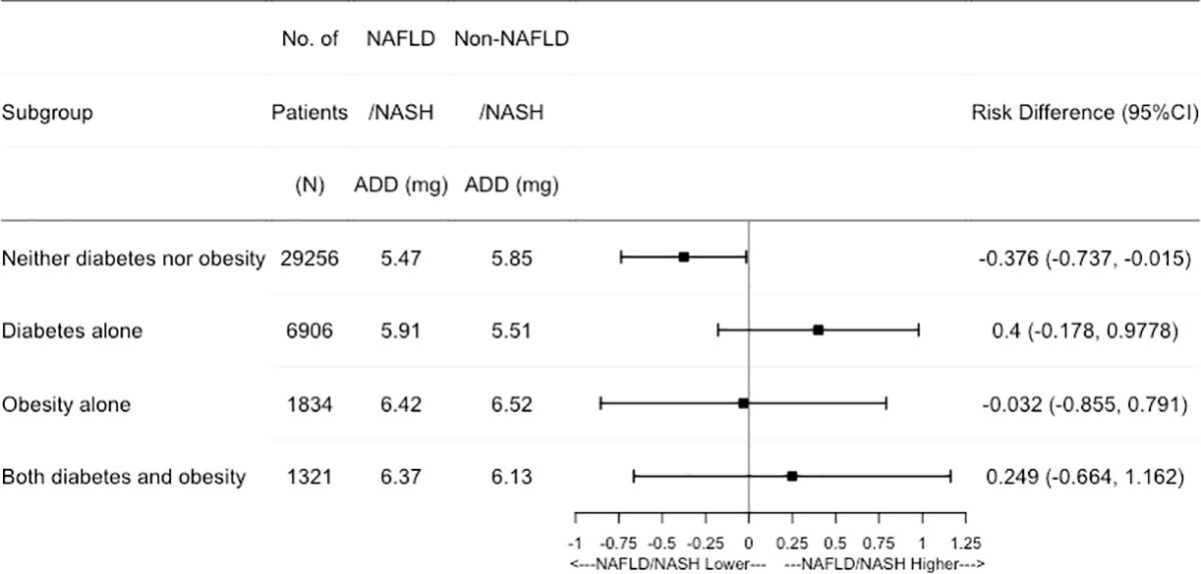
Adjusted effect estimate of NAFLD/NASH on average daily dose of warfarin using the propensity score stratification approach after the first 2 months of warfarin use. Note: NAFLD: Nonalcoholic Fatty Liver Disease; NASH: Nonalcoholic Steatohepatitis; ADD: Average Daily Dose.

Results from subgroup analyses show that NAFLD/NASH patients without obesity and diabetes tended to have a significantly lower average warfarin daily dose compared to patients without NAFLD/NASH to attain therapeutic range of INR (RD: -0.38; 95%CI: -0.74–-0.02) (Fig 3). In the subgroup of patients with obesity, diabetes, or both, there are no significant differences of warfarin dose between NAFLD/NASH and non-NAFLD/NASH patients (Fig 3). Among patients with diabetes alone, there is a tendency that patients diagnosed with NAFLD/NASH might require higher warfarin dose though the difference fails to reach statistical significance.

**Fig 4:**
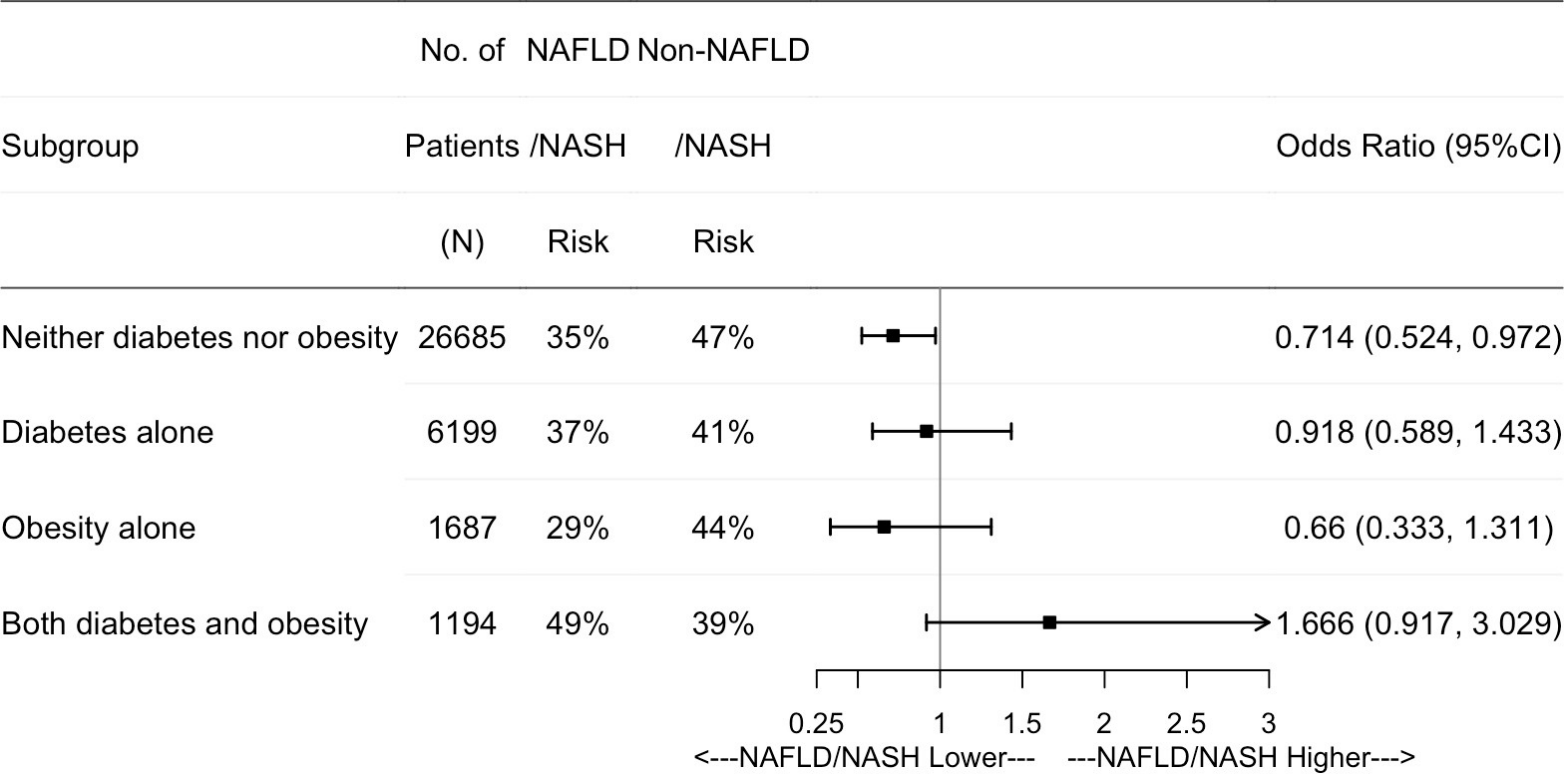
Adjusted odds ratio of TTR>60% among NAFLD/NASH vs Non-NAFLD/NASH using the propensity score stratification approach after warfarin initiation. Note: NAFLD: Nonalcoholic Fatty Liver Disease; NASH: Nonalcoholic Steatohepatitis; TTR: Time to Therapeutic Range.

Further subgroup results show that NAFLD/NASH patients without obesity and diabetes have a significantly lower odds of achieving TTR>60% compared to patients without NAFLD/NASH (OR: 0.71; 95%CI: 0.52–0.97) (Fig 4). In the subgroup of patients with obesity alone or with diabetes alone, there were no differences in having TTR>60% between NAFLD/NASH and non NAFLD/NASH patients (Fig 4). In the subgroup of patients with both diabetes and obesity, NAFLD/NASH is related to an increased odd of TTR>60% compared to non-NAFLD/NASH but does not achieve statistical significance (OR: 1.67, 95%CI: 0.92–3.03) (Fig 4).

## Discussion

In this study, the mean warfarin ADD in patients diagnosed with NAFLD/NASH was 5.853 mg, and 5.848 mg in patients without NAFLD/NASH. Using GLM with PS fine stratification with main effects only (no interaction terms), no significant effect of NAFLD/NASH was observed on either warfarin dose or TTR>60% (S1 Table). However, our study demonstrates that patients diagnosed with NAFLD/NASH required a lower warfarin average daily dose to obtain and maintain therapeutic range of INR relative to patients without NAFLD/NASH in patients without a diagnosis of obesity or diabetes, which indicates that obesity and diabetes are two potential effect modifiers that alter the effects of NAFLD/NASH on warfarin dose. Several studies have found that obese patients required a higher ADD of warfarin and a longer time to achieve therapeutic INR compared with normal-weight patients because of increased absolute volume of distributions [12,47–49]. Considering the high prevalence of NAFLD/NASH in obese patients, ranged from 50% to 90% [21], warfarin dose reduction in NAFLD/NASH patients could be cancelled out by the increased warfarin dose in patients with both obesity and NAFLD/NASH. Thus, significant differences between patients with or without NAFLD/NASH weren’t observed in obese patients or overall study cohort that has high prevalence of both obesity and NAFLD/NASH.

Furthermore, our analysis results show that NAFLD/NASH resulted in 28% of decreased odds of achieving TTR>60% in patients without obesity and diabetes, while in patients with both obesity and diabetes, it is related to 95% increased odds of having TRR>60%. Similarly, significant effects were not observed in patients with only obesity or diabetes. It has been noted that patients with chronic liver disease had a lower average TTR and increased risk of hemorrhages relative to those with no chronic liver disease [50]. However, these studies focused on more advanced liver diseases, not including NAFLD/NASH. Previous study results for obesity and TTR are controversial. One study reported that obesity was associated with lower TTR and deteriorated anticoagulation quality [51], while a post hoc analysis using clinical trial data showed that obese patients had higher odds of achieving TTR≥60%, and were related to a better quality anticoagulation control [52]. In our study, obesity is independently related to lower odds of TTR>60% (S1 Table).

We have shown that a significantly lower ADD of warfarin is required to maintain INR within the therapeutic range of 2-3 in patients diagnosed with NAFLD/NASH but without diabetes and obesity. Clearance of warfarin from the body is dependent on the expression and activity of hepatic Cytochrome P450 enzymes including CYP2C9 and CYP3A4. Clinical and experimental data from our research group and others has shown considerable dysregulation of several pathways of drug metabolism in both diabetes and NAFLD/NASH patients [22,53]. For example, we have shown that the expression and activity of cytochrome P450 (CYP) 3A4 which is responsible for the biotransformation of 55% of marketed medications is significantly reduced in livers from donors with diabetes and NAFLD/NASH [53]. Moreover, we have shown a reduction in the CYP2C9 protein and mRNA expression in liver from NAFLD/NASH patients and in vitro models of NAFLD (manuscript in preparation). Therefore, we have hypothesized that NAFLD/NASH is likely to impact the warfarin dose required to achieve a therapeutic INR level. In the current analysis, the higher warfarin ADD in patients diagnosed with NAFLD/NASH is in concordance with the reduced warfarin clearance in such patients. However, the effect of other factors including obesity and diabetes on both pharmacokinetics and pharmacodynamics of warfarin cannot be excluded.

NAFLD/NASH is strongly associated with T2DM, for which insulin resistance plays an important role in the pathogenesis of NAFLD/NASH [54,55]. Approximate 60% of T2DM patients have NAFLD/NASH [56]. A significant bidirectional relationship between NAFLD and T2DM/Metabolic syndrome has been observed [57,58]. A meta-analysis study revealed that warfarin is associated with higher risk of stroke or systemic embolic events, intracranial hemorrhage, all-cause mortality, and gastrointestinal bleeding in atrial fibrillation patients with diabetes [59]. The effect of T2DM on warfarin dose hasn’t been extensively investigated yet, and concerns remain that warfarin may be less favorable than direct oral anticoagulants (DOACs) for patients with diabetes and AF due to increased risks of calcification in coronary and renal artery resulting from reduced matrix Gla protein [60,61]. The use of DOACs will exceed that of VKA even in liver disease patients. Historical cohorts of patients with liver disease are still anticoagulated with VKA but a growing mole of data on the use of DOACs in patients with advanced liver disease is accumulating. Although patients with active liver disease have been excluded in all the pivotal RCTs of DOACs, real-life data suggest that these drugs are as much effective and maybe safer in liver disease patients as compared to those without liver disease. In our sensitivity studies, diabetes, along with obesity, appear to be significant effect modifiers that alter the effect of NAFLD/NASH on either warfarin dose and quality anticoagulation control (TTR>60%). In patients with neither diabetes nor obesity, NAFLD/NASH is related to an inferior anticoagulation, while in patients with both diabetes and obesity, NAFLD/NASH is not associated with quality anticoagulant control. This could be because the relationship of NAFLD/NASH and quality anticoagulant control is distorted due to the number of patients in this subgroup.

Clinical factors have been proven to explain partial variability of warfarin therapeutic dose. In BF Gage et al study, clinical factors were estimated to account for 17-21% of the variability in the warfarin therapeutic range [8]. In this study, we have adjusted for several demographic and clinical risk factors as covariates in the multivariable model. These predefined covariates have been identified and fitted in published algorithms as significant predictors for warfarin daily dose [8,9].

It has been confirmed in basic laboratory studies that the liver plays an essential role in both synthesis of coagulation factors and metabolism of anticoagulant drugs [62,63]. Warfarin undergoes 100% of hepatic metabolism, thus, lowered liver function could affect the metabolism and effect of warfarin [62]. In clinical practice, anticoagulation abnormalities have been reported in patients with liver disease [62]. In addition, studies suggest a strong association between chronic liver diseases and a higher risk of venous thrombotic complications [62–64]. Therefore, our study results showing a reduced warfarin daily dose and lower odd of TTR>60% in NAFLD/NASH patients may relate to the anticoagulation abnormalities caused by hepatic dysfunction.

It has been noted that anticoagulation control varies markedly between warfarin new users and stabilized users [65]. The INR stabilization was defined as the first 3 consecutive INR values within 2.0 and 3.0 after warfarin initiation [45]. Approximately 61,337 patients (70%) failed to reach INR stabilization. After initial INR stabilization, 44% of INR values were out of the target range of 2.0-3.0.

We only included INR measures within therapeutic range among stabilized warfarin users in the sense that subtherapeutic dose of warfarin were eliminated. Several medical conditions may be associated with warfarin metabolism. In a previous study, among patients treated with phenprocoumon, patients with a greater BMI required a longer time to attain target INR. In addition, these patients also required a higher cumulative dosage of phenprocoumon until a therapeutic INR was attained [66].

There are a number of limitations in this study. First, the propensity score method only addressed all measured confounding factors. Unmeasured confounding factors, such as genotype, smoking, diet, and race that are not included in the claims data, may bias the results in a way that we cannot anticipate. Although liver function is important, Glutamate-transpeptidase and glutamic-oxalacetic transaminase are not included in the data. Based on the guideline for pharmacogenetics-guided warfarin dosing by Clinical Pharmacogenetics Implementation Consortium, CYP2C9, VKORC1, CYP4F2, and rs12777823 genotypes can be used to estimate initial warfarin dose to achieve an INR target of 2-3 [67]. However, these genotypes may not relate to the pathogenesis of NAFLD/NASH, therefore, not on the causal pathway of NAFLD/NASH and warfarin dose. Second, TTR was calculated using linear interpolation (Rosendaal’s approach), in which the last INR measure was assigned to subsequent days without measured INRs through a linear plot [44]. Only stabilized INRs were included in the calculation of TTR. INRs that were sub- or supratherapeutic were not used in linear interpolation. Third, there were a portion of INR lab measures unreported in Optum database. We assumed missing is at complete random, thus, didn’t impute any missing INR values in this study. Fourth, NAFLD/NASH is an underrecognized epidemic due to lack of symptoms and diagnosis test [68]. Using diagnosis codes to identify NAFLD/NASH patients in claims data might not allow to know the functional Child-Turcotte-Pugh score of the liver disease and underestimate NAFLD/NASH patients, which results in small sample size and conservative findings. The last, adherence could be a potential risk of selection bias as warfarin is typically indicated for long-term use. Those patients who took warfarin in a short period were excluded from the study cohort.

To our knowledge, this is the first study to evaluate whether a similar phenomenon exists with warfarin as evaluated by INR and NAFLD/NASH in patients. Clinicians may find it useful when initializing warfarin to consider a combination of comorbidities that may interact with warfarin.

## Conclusions

Our study identified the interaction effect of NAFLD/NASH with obesity or diabetes on warfarin dose prediction to obtain the INR therapeutic range of 2-3. NAFLD/NASH is related to a lower daily warfarin dose and decreased odds of having TTR>60% in patients without obesity and diabetes. Further investigation with a larger sample size, and a stronger study design, either a multicenter prospective cohort study or a multicenter randomized clinical trial, is warranted.

## Supporting information

S1 Table Adjusted Difference according to NAFLD/NASH, Obesity, and Diabetes: Average Daily dose of Warfarin and TTR >60% After the First 2 Months of Warfarin Use, using PS Strata Exposure Approach. Note: OR: odds ratio of TTR > 60% among patients with NAFLD/NASH vs patients w/o. NAFLD/NASH. Statistically significant values are indicated in bold. Adjusted for age, insurance type, indications of warfarin, comedication and preexisting conditions presented in Table 1. NAFLD: Nonalcoholic fatty liver disease; NASH: Nonalcoholic Steatohepatitis; ADD: Average Daily Dose; TTR: Time to Therapeutic Range.
(PDF)
