# Supplementary material for: Required warfarin dose and time in therapeutic range in patients with diagnosed Nonalcoholic Fatty Liver Disease (NAFLD) or Nonalcoholic Steatohepatitis (NASH)
Source: PLoS One. 2021 Sep 15;16(9):e0251665. doi: 10.1371/journal.pone.0251665 (PMC8443040; doi:10.1371/journal.pone.0251665)
Supplement: S1 Table — Note: OR: odds ratio of TTR > 60% among patients with NAFLD/NASH vs patients w/o. NAFLD/NASH. Statistically significant values are indicated in bold. Adjusted for age, insurance type, indications of warfarin, comedication and preexisting conditions presented in Table 1. NAFLD: Nonalcoholic fatty liver disease; NASH: Nonalcoholic Steatohepatitis; ADD: Average Daily Dose; TTR: Time to Therapeutic Range. (PDF) [file pone.0251665.s001.pdf]

**Outcomes**	**Parameter**	**Dose difference**	**95% CI**	**P-value**
Warfarin ADD	NAFLD/NASH	-0.02	(-0.31, 0.26)	0.8660
	Obesity	**0.68**	**(0.51, 0.85)**	**<0.0001**
	Diabetes	**-0.15**	**(-0.26, -0.03)**	**0.0103**
TTR >60%	**Parameter**	**OR**	**95% CI**	**P-value**
	NAFLD/NASH	0.88	(0.75, 1.09)	0.2505
	Obesity	**0.88**	**(0.77, 0.98)**	**0.0281**
	Diabetes	**0.81**	**(0.74, 0.88)**	**<0.0001**
